# Characteristics of Large Antlers Reveal Key Features to Reach Full Genetic Potential

**DOI:** 10.3390/ani15091303

**Published:** 2025-04-30

**Authors:** Tomás Landete-Castillejos, Irene Arroyo, Martina Pérez-Serrano, Dainis Paeglitis, Mara Paeglite, Louis Chonco, Andrés J. García

**Affiliations:** 1 Sección de Recursos Cinegéticos y Ganaderos, Instituto de Desarrollo Regional (IDR), Campus Universitario s/n, 02071 Albacete, Spain; irene94ab@gmail.com (I.A.); louis.chonco@uclm.es (L.C.); andresjose.garcia@uclm.es (A.J.G.); 2 Instituto de Investigación en Recursos Cinegéticos (IREC), Consejo Superior de Investigaciones Científicas, Universidad de Castilla-La Mancha-Junta de Comunidades de Castilla-La Mancha (CSIC-UCLM-JCCM), Ronda de Toledo s/n, 13071 Ciudad Real, Spain; 3 Departamento de Ciencia y Tecnología Agroforestal y Genética, Escuela Técnica Superior de Ingenieros Agrónomos y de Montes y Biotecnología, Universidad de Castilla-La Mancha (UCLM), Campus Universitario s/n, 02071 Albacete, Spain; 4 Departamento de Producción Agraria, Escuela Técnica Superior de Ingeniería Agronómica, Alimentaria y de Biosistemas, Universidad Politécnica de Madrid, 28054 Madrid, Spain; martina.perez@upm.es; 5 Saulstari Deer Farm, More Parish, Sigulda District, LV-2170 Sigulda, Latvia; deerparks@inbox.lv (D.P.); deerevent@gmail.com (M.P.)

**Keywords:** deer antlers, bone size and structure, secondary sexual characters, red deer, resource investment

## Abstract

Antlers are costly bone structures that grow every year. Thus, in addition to the genetic potential, its full potential size depends on the physiological state and food availability. Our aim was to examine antler characteristics related to genetic potential (burr perimeter) with final antler investment (antler weight) and how they relate with antler structural characteristics: beam length, circumference at mid and upper parts of the beam, and length of first (brow) and midbeam (third) tines. We measured 206 antlers from the University of Castilla-La Mancha experimental farm (UCLM) and from 203 top size antlers from Latvian International Antler Competition (LIAC). The results show that UCLM and LIAC had antlers of similar length (4.6% difference), but LIAC had 47% heavier antlers. The body growth explained that correlations of antler measurements with age were higher in males of 3 or more years, but much weaker in fully grown males (5 years or older). Weight reflected investment in antlers, as it showed the greatest correlation with all measurements, but length had weaker correlations. A large burr cross-section seems to reflect genetic potential, because it is necessary but not sufficient to grow large antlers. The results could be a useful tool for deer breeders.

## Simple Summary

Large antlers are important in red deer breeding and hunting, but they are also fighting structures in which males can invest heavily (up to 28% of their skeleton material, which is mobilized for its fast growth). Deer could grow fighting antlers that are larger and weaker, shorter and more robust, or their antlers could be on a scale of non-variable patterns. In addition to the genetic growth potential, food availability and health state or senescence of the male could affect how the different parts of the antler are sequentially grown. In this study, we examined how the different parts of the red deer antler correlate with each other and with variables that could estimate antler investment (weight or length) in a set of antlers from males fed a balanced diet from farms. Age influenced antler characteristics only if still-growing males were included (3 and 4 years). Weight is the best estimate of investment in antlers, as it showed a higher correlation than length with the rest of antler characteristics. Burr (the base of the antler where it connects to the head of the deer) seems to show the growth potential, as large antlers had large burrs, but the antler could be shorter or lighter than predicted, likely if other factors reduced the potential antler investment.

## 1. Introduction

As is often the case with other sexual characters [1], antlers are a costly character and are directly related to fighting ability, access to females, and position in the dominance hierarchy [2,3,4]. The fact that antlers are costly to grow is reflected in the fact that they are the fastest growing tissue in any animal: from 1 to 4 cm/d in length, and producing more than 20 cm^2^/d of skin at the tips, where they grow [5], and they have adapted their cell biology to produce such astonishing full regeneration [6]. Thus, if antlers are costly to grow, as it is reasonable to expect, antler size depends on the interaction between the heritability [7] and a strong influence of environmental conditions [8,9,10,11,12]. These environmental conditions include food availability, both in the past and present, as it can lead to greater body weight (partly evolved from a high body growth trajectory). Thus, the allometry antler size vs. body size, is one of the oldest known allometries in science ([13]; based on data from the XIX century). Schröder proposed a relationship between antler weight and body weight, with two different coefficients depending on whether the animals were young or older than 5 years [14]. Gómez et al. [15] also found a different investment between still-growing deer, which could invest from 6 to 23% of the skeletal weight in antlers (respectively, 1 to 3 years old), and older ones, which could range from 30 to 35% (4 and 5 years old, respectively). Although performed in a farm to control all variables, this study is not different from studies in the wild in Italy [16] in which antler mass grows up to 5–6 years of age, then reaches a plateau up to 11 years of age, and subsequently is reduced for senescence (a condition that is not allowed in farms as it is the case of the data included in this study). The age and quality or availability of resources for antler growth not only affect antler size: in the largest sample size (244) over the longest study period (17 years), Gomez et al. [17] found that older and heavier males cast their antlers earlier and grow them faster than younger and lighter males, probably to synchronize subsequent antler growth with the spring peak in plant nutrients.

Considering that antlers reflect the enormous physiological effort required to grow them [5], it is not surprising that the deterioration of the physiological machinery caused by senescence is translated into smaller antler size [18]. In addition to the general effect that reduced nutrient availability in the antler can have, the effect is more pronounced in the last section grown (the upper part) as the availability of minerals and other bone materials is closer to depletion [19,20]. Resource depletion affects both composition, mechanical properties (the latter references) and even histology [21].

**Figure 1:**
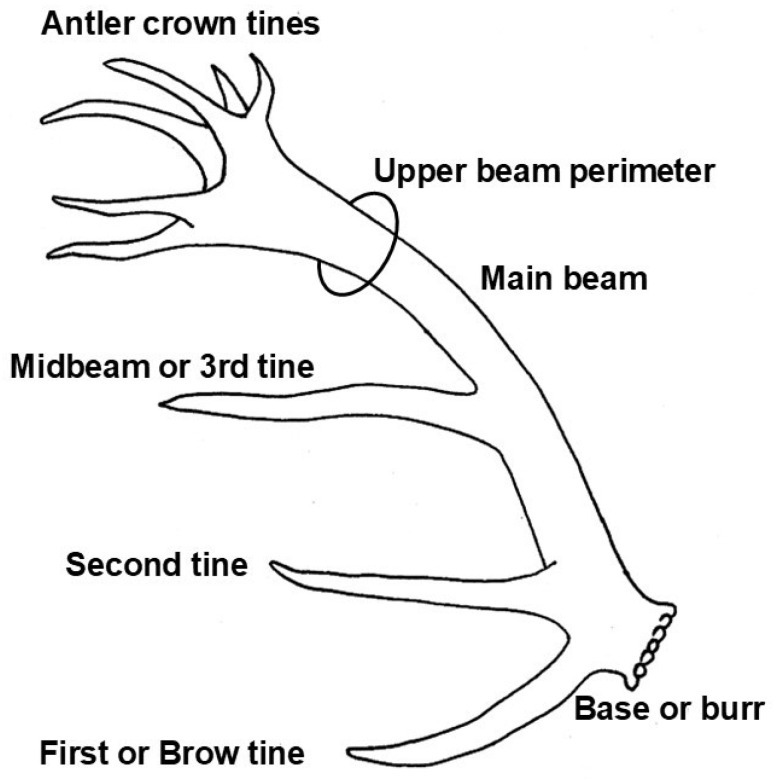
Drawing of red deer antler with main antler characteristics used in this study: length of brow or first tine and that of third one, upper beam perimeter (as well as that of burr), length of main beam, and number of tines in the crown. Drawing made by I.A.

Thus, the previous findings suggest that the external structure of antlers should be influenced by the interaction between genetic potential (which could be estimated as the burr cross-section-measured as circumference- and what we consider, in turn, an estimate of the potential blood flow with nutrients to grow larger or smaller antlers), and the availability of resources to grow them. The interaction between these factors should produce not smaller or larger antlers with the same scale of relationships between different parts, but a variation between antler parts (variation in the correlations between antler length and that of the first or midbeam tine, perimeters at the base of the first and second third of the antlers, etc.), which could provide information about the priorities in the antler growth. We therefore set out to assess how the availability of resources to grow an antler (estimated as its weight) varies with antler length, burr circumference (estimate of its cross-section) and those at different beam lengths, the length of the brow (first) or midbeam (third) tine, and the number of tines in the crown (Figure 1). For this, we used an outstandingly large set of 409 antlers both from the most important international antler competition in Europe (which is organized in Latvia: the Latvian International Antler Competition, LIAC) and from the UCLM experimental deer farm. Id est, it can be assumed that the deer are in good condition in all cases, although differing in genetic potential (as well as age), and probably with minor variations in nutrition quality between the different farms taking part in the competition. Age is also an important factor, as the sets include relatively young animals that may still be gaining weight (3–4 years of age) and those that have reached their full size (5 years or older). The effect of age is likely to be more influential in the antler characteristics in the first group (considering that the neither the antler competition nor the UCLM experimental farm include senescent males, in which case age would again become important again as a factor influencing antler characteristics). The data set is not adequate to show the effects of senescence in very old animals, despite having 13% antlers from males aged 9 years or more at LIAC (see below) because old animals in farms are only kept if they are in good condition to mate and having extraordinarily big antlers.

We consider these as the main hypotheses: (1) when all males are included (3 years or older), the correlation between age and antler characteristics should be higher than when prime age males are considered (5 years or older). The reason is that males of 3 and 4 years of age are still using resources to grow their bodies, whereas after 5 years of age, age should not be more important than genetic differences in determining antler size or weight. (2) Burr perimeter, which is an estimate of burr cross-section, reflects the genetic potential to grow large antlers. However, as the final antler size depends on the availability of body resources (in turn, related to nutrition and health state), heavier antlers (reflecting a greater amount of resources to grow large antlers) should be more influenced (e.g., higher correlation) by antler length, beam perimeter near the crown, or length of the midbeam tine, than by burr perimeter or length of the brow/first tine. (3) Since genetic potential in the form of burr cross-section can only be maintained or reduced, the main source of variation or explanatory power (in GLMs) of beam perimeter along the beam should be the burr perimeter rather than beam or tine length.

## 2. Materials and Methods

### 2.1. Experimental Design

The animals were measured according to the Trophy Evaluation System (TES) of the International Council for Game and Wildlife Conservation (CIC for its acronym in French). The measurements were taken as follows: **Antler length**, measured following the outside curvature from the lower outer edge of the burr to the tine of the crown that produces the longest measurement. We took the average of both antlers in the set, as with other variables. **Length of the brow or first tine**, measured on the front-lower side, from the burr to the end. **Length of the midbeam (third) tine**, measured in the same way, but taken from the junction with the main beam to the tip. **Burr perimeter**, measured by a simple circular extension of the gauge over the thinnest part of the burr. The value used is, again, the average of both left and right antlers. **Lower and upper perimeters of the antler beam**, measured in the section of the thinnest part between the brow tine and the midbeam tine for lower beam perimeter, and between the midbeam tine and the crown for upper beam perimeter. We also measured **antler weight** as the weight of each of the antlers of the set added (balance precision ± 10g). In all cases, separate antlers without the skull were used, so that there was no need to estimate the weight of the skull and subtract it from the set of antlers and skull. **Number of tines in the crown** counted as those measuring at least two centimeters at the shortest point. All points above the central point are part of the crown.

The age of animals in UCLM experimental farm and the LIAC are shown in Table 1. A total of 409 antlers were used, from which 206 belonged to UCLM and 203 were trophies from LIAC. From these, 193 were males still growing of 3–4 years (UCLM = 109, LIAC = 84) whereas 216 males were in prime age (UCLM = 97, LIAC = 119).

### 2.2. Statistical Analysis

For the statistical analysis, a database was created with all the trophy homologation measurements together with the age of the animal, the origin of the animal (Latvian competition or UCLM), and the identification of the animal if it had one. Although data from animals kept under optimal conditions in a farm could be considered as not very representative for the conditions in the wild, some of the most valued classical studies [22] only used data from exceptionally large-antlered males because Geist (pg. 183) considered them as more biologically meaningful for taxonomic purposes. These are the same type of trophies as are used in the current study. For a given individual, the mean of left and right antlers was computed for all measurements. When the value of one side was missing, we used the measurement of the only antler available as the average for both.

One-way least-squares analyses of variance (ANOVAs) were conducted to compare the antler measurements for LIAC with those for UCLM experimental deer farm, both for prime age stags with 5 years of age or older. In addition, in order to assess the relationships between antler measurements and all of them with age, we calculated Pearson correlations between pairs of variables, both using all data from all adults (3 years or older, upper value), or only males in prime age and physiological state (5 years or older, lower value in bold). Finally, a general linear model (GLM) test was performed to assess males of 5 years or older, which antler variables were the most influential, rendering the others as non-significant. In addition, the statistic eta square provided an estimate of which variable had the greatest influence on the antler measurements subjected to the GLM (eta square ranges from 0, or no influence, to 1, maximum influence). The variables studied were antler weight, beam length, upper beam circumference, and number of tines. Values are given as means and standard error of mean. Differences were considered significant at *p* < 0.05. All analyses were carried out with SPSS version 22.0 (2013) (SPSS Inc., Armonk, NY, USA).

## 3. Results

The comparison in large antlers from prime age males (5 years or older) between the LIAC and UCLM Experimental Deer Farm (UCLM) shows, unsurprisingly, that antlers in the international competition gathering the best antlers from all Europe were larger than those of the UCLM Deer Farm. It is rather surprising that the difference, although significant, is not very large in length (4.6%; *p* < 0.05): 90.3 ± 1.1 cm from LIAC vs. 86.3 ± 1.6 cm from UCLM. The difference is larger in weight (47%, *p* < 0.001): 7850 ± 0.270 kg (LIAC) vs. 5.340 ± 0.250 kg (UCLM). The rest of the means can be seen in Table 2. The difference is also high in brow (first) tine length (31% bigger for LIAC), in midbeam or third tine (43% bigger in LIAC), somewhat smaller in burr circumference (14% bigger in LIAC), 20% and 21% for lower and upper beam circumference, respectively (both bigger for LIAC). The greatest difference was for the number of tines of the antler: 77% bigger for LIAC as compared to UCLM.

Despite the differences, and in order to increase sample size, data from both origins were pooled to analyze biological effects on antler size that, in general, correspond to males in prime physiological state.

In order to assess the interrelation between antler characteristics of adult males including those that have not reached their maximum size (3 years or older, an age in which they are still gaining weight) and those of peak physiological state (5 years or older), we show in Table 3 the correlations between antler characteristics in both age groups (all adults, vs. prime state ones: 3 years or older vs. 5 years or older). The first interesting difference between both types of correlations is that age shows stronger relationships with all antler characteristics when all adult animals are included, compared to when only animals of 5 years or more are included (52.9% higher for 3 or more years than for 5 years or over). This may be expected as the variability in antler characteristics is greater in younger animals. However, the second surprising fact is that, apart from correlations with age, the rest of the correlations between variables are higher when only prime age animals are included in the analysis (21.8% higher for prime age males than for those of 3 years or over).

The GLMs of antler characteristics are shown in Table 4. Antler weight could be considered an estimate of the resources placed in growing the antler (in terms both of minerals and the protein involved). The model shows a very large proportion of variability explained for this sample size: R^2^ = 82%. The antlers with a greater amount of resources placed on them (i.e., greater weight) were those that had a longer beam, a longer midbeam tine, and a bigger cross-section (measured as circumference) of burr and upper beam. Surprisingly, the length of brow or first tine did not influence antler size once the midbeam tine was included. Contrary to what could be expected in terms of factors influencing mostly the weight of the antler, it is not the burr section or beam length which were the most influential (despite all blood and mineral flow has to cross the burr), but, as revealed by eta^2^, the most important factor was how large the upper beam section was (measured as circumference length: eta^2^ = 0.30), followed by midbeam tine length (eta^2^ = 0.21), with beam length having a similar size of effect (eta^2^ = 0.19), and burr section having the smallest effect (eta^2^ = 0.05). Age did not significantly affect the resources placed in the antler (i.e., its weight) once the animals reached 5 years or older.

Regarding antler length, in contrast, age had a significant influence (*p* = 0.013), although it was one of the smallest effects observed (eta^2^ = 0.03). The factor having the greatest influence was, unsurprisingly, antler weight (as an estimate of the resources a prime age male can put into growing a large antler according to its full genetic potential; eta^2^ = 0.21). Second to this effect, at a rather large distance, was the upper beam cross-section (measured as circumference: eta^2^ = 0.09), and the length of the midbeam (third) tine (eta^2^ = 0.02).

With regards to upper beam circumference, it is not surprising that its cross-section (directly related to circumference) depends upon burr circumference (because the cross-section along the length of the antler can only be equal or less than that on the base or burr). A striking finding is that antler weight, a measure of the available resources to maintain a beam cross-section as large as that of the burr, is not significant. No other antler variable was significant in this model.

Finally, the number of tines, most of which are in the crown, mainly depended, unsurprisingly, on variables that are directly related to the amount of resources that encourage an antler to continue growing (upper beam circumference and length of midbeam tine: eta^2^ = 0.23 and 0.20, respectively). The length of the antler was much less influential. Surprisingly, after considering these variables, antler weight was not significant.

## 4. Discussion

The results of this study have a double value: on the one hand, they indicate what variables are truly related to the capacity of antlers to reach their full genetic potential (e.g., those that, having a large burr or cross-section at the base, will later become very large antlers). On the other hand, the results are also a tool that can be used by deer breeders who want to select for large antlers, and they can select animals with high genetic potential for large antlers (e.g., based on the burr cross-section) and later detect if they fail to reach this potential (i.e., if the circumference at the lower or upper antler beam is significantly lower than at the burr, which may indicate health or nutritional problems).

The first interesting finding is the comparison between antlers in UCLM experimental farm, and those in the LIAC. Both antlers differ only 4.6% in length (bigger in LIAC). However, LIAC antlers are 47% heavier than those in UCLM. Many deer breeders and hunters think that both are a measure of the same thing: the amount of resources that the deer invest in antlers. However, correlations show that both are not correlated when all adult males are included, and for prime age males (5 years or older), the correlation is only R = 0.55, whereas for the same prime age males, the correlation of antler weight and another measure of the beam, the upper circumference, is 44% bigger (R = 0.80). This shows that antler weight is a much more reliable measure of the investment in antlers than antler length. This, in itself, is a useful tool for breeders and hunters.

One may think that the reasons for this difference between UCLM and LIAC antlers lie in the fact that the first is an experimental farm, and the other is an antler competition with the best deer bred for antlers. This partly true, but Table 1 also shows that age structure may have influenced the difference UCLM-LIAC: 13.4% of the LIAC antlers were from males aged 9 years or older and still in prime condition, whereas only one male from 97 in UCLM had more than 9 years. In a farm, animals this old are only kept if they are particularly good trophies, therefore contributing with particularly heavy antlers to the data set.

The question then arises: where does the difference between the 47% greater weight for LIAC and only 5% longer antlers go? The results show that the remaining weight difference is invested in growing larger main tines (midbeam or third tine 43% bigger in LIAC than in UCLM farm, brow tine 31% bigger), and also those in the crown or top (23 vs. 13 tines in LIAC and UCLM, respectively). Obviously, having mid and upper beams in LIAC around 20% bigger than in UCLM antlers in circumference (related to cross-section) means that, if devoid of tines and cut to a standard size below the crown, the beam of LIAC antlers would be heavier than an equal size-only beam of UCLM university.

The same results can be shown with another statistical parameter: the correlations. Whereas antler weight (or amount of resources placed in antler) is related with beam length by R = 0.55, weight is related with burr, lower beam or upper beam circumference about 50% more (R = 0.77, R = 0.76 and R = 0.80, respectively). Equally, weight is correlated with midbeam tine length (R = 0.74) or number of tines in the crown (R = 0.61) much more than with antler length.

Another interesting fact that goes against the widespread consensus among deer breeders is that, regarding the most useful indicator of quality of the antler (i.e., antler weight), the most influencing factor is not burr section (despite all blood and mineral flow has to cross the burr), but, as revealed by eta^2^ in the GLMs, the most important factor was how large the upper beam section and midbeam tine length was (which are six and four times more influential than burr section). Burr perimeter (estimate of cross-section) has the smallest effect on antler weight. Also, contrary to the belief of many deer breeders, age was not even significant in the model once the antler variables were included and the animals were in their prime age. The most likely explanation is that burr section could be considered as having the potential to grow larger antlers, like how a big pipe can allow a big flow of, for example, water. However, the amount of resources that are transferred will depend on other factors such as the physiological state of the deer (e.g., if he is sick when the antlers are growing, or if he has a good amount of food or not), i.e., a big pipe may only carry a small flow of water despite its size. However, if the antler has a big cross-section in the upper part, it shows that the antler has grown with a greater number of resources to achieve a larger size. The burr shows the potential of growing a large antler and is more or less the same every year, so that such potential is only reached if the deer has a high number of resources that particular year to be mobilized in order to grow a large antler. Equally, the three main tines (midbeam) can only grow large in size if the antler is big. If it is small, the first tine could still be rather large, so this is not a good predictor of antler weight.

The GLM on beam length shows that, by far, the most influential factor is the amount of resources available to grow a large antler: antler weight (apart from upper beam circumference, the other factors are 7–10 times smaller in importance than antler weight). In other words, it is possible to have heavy antlers that are not necessarily long, but it is not possible to have long antlers if they are not heavy (and with large main and crown tines). This provides a more detailed picture, with subtle differences compared to the popular knowledge among breeders and hunters.

The GLM for upper beam circumference is also interesting. As can be seen in the correlations in Table 3, upper beam cross-section (i.e., circumference) has a high correlation with antler weight and lower beam circumference than that of burr. However, in the GLM, the only significant factor that can influence the upper beam circumference, once all the factors in the correlations are taken into account, is the burr cross-section (i.e., its circumference). In other words, it is not possible to have a large cross-section in the upper parts of the antler that it is not equally large in the base. This means that the pipe, which is the antler, can only remain the same as in the base (only if there are enough resources to maintain the flow of minerals and proteins).

In summary, the current study shows the important factors influencing antler investment with a unique set of antlers from many European countries, all of them grown in a situation of high availability of food and optimum health. Age is only important if adult animals are included in the set when they are still reaching their prime age (before 5 years of age). Once males are in their prime life period, age is not often significant in explaining variability in antler traits. Weight is the best estimate for antler investment, as all the antler parts grown add weight. A large burr cross-section is necessary but not sufficient to grow large antlers, as it sets a limit on how much the antler can grow, but this potential can only be reached depending on the available resources for a particular year. However, if the cross-section can be kept in upper parts without serious decrease in cross-section, then it means that the antler will be large, and all the different parts will also be big. The results can be a useful tool for deer breeders in addition to showing structural relationships among the different antler parts.

## 5. Conclusions

Antlers do not grow in a fixed pattern that results in correlations between all parameters close to one (i.e., only varying in scale) but instead show a variability among different antler traits that conveys information about the growth priorities of different traits. Age only influences antler traits, i.e., shows a higher correlation, when adult males of 3–4 years of age that are still gaining weight are included. This suggests that part of the resources at these ages are invested in skeletal growth rather than on the antler growth. The best parameter to estimate the investment in larger antlers is weight rather than length, as heavy antlers result in higher values of all measured parameters, but this is not necessarily true (i.e., lower correlation) with antler length. Burr cross-section is an estimate of the genetic potential to grow large antlers, as it sets the limit for growing heavy antlers (large upper beam cross-section), but other factors may reduce this potential (i.e., heavy antler weight is less correlated with burr cross-section than, for example, middle, and upper beam cross-sections).

## Figures and Tables

**Table 1:** Age composition of the data used in the study.

		Antler Origin	
		UCLM	LIAC
Age (years)	3	61	34
	4	48	50
	5	41	29
	6	32	32
	7	15	21
	8	6	9
	9	2	12
	10	0	9
	11	0	3
	12	0	2
	13	1	2
Total		206	203

**Table 2:** Comparison of antler measurements between Latvian International Antler Competition and UCLM experimental deer farm, both for prime age stags (5 years or older).

	LATVIA	UCLM University Farm	
	Mean ± SEM	Mean ± SEM	Sig.
Beam length	90.3 ± 1.1	86.3 ± 1.6	*
Antler weight	7.9 ± 0.3	5.3 ± 0.2	***
Brow (1st) tine length	38.3 ± 0.7	29.2 ± 0.7	***
Midbeam tine length	41.8 ± 0.7	29.2 ± 1.0	***
Burr	27.0 ± 0.3	23.7 ± 0.4	***
Lower beam circf.	17.4 ± 0.2	14.5 ± 0.2	***
Upper beam circf.	16.8 ± 0.3	13.9 ± 0.2	***
Num. tines	22.9 ± 0.6	13.2 ± 0.4	***

Probability levels as follows: * for *p* < 0.05; *** for *p* < 0.001.

**Table 3:** Correlations between antler characteristics showing, for each cell, data from all adults (3 years or older, upper value), and prime physiological state (5 years or older, lower value in bold).

	Age	Antler Wgt	Beam Length	Brow T Length	Midbeam T Length	Burr	Lower Circf	Upper Circf
Antler wgt	-							
	0.356 **							
Beam Length	0.506 **	-						
	0.265 **	0.551 **						
Brow T Length	0.568 **	0.106 *	0.533 **					
	0.395 **	0.536 **	0.448 **					
Midbeam T Length	0.482 **	0.138 **	0.466 **	0.705 **				
	0.369 **	0.744 **	0.452 **	0.637 **				
Burr	0.502 **	-	0.551 **	0.588 **	0.657 **			
	0.314 **	0.771 **	0.524 **	0.495 **	0.651 **			
Lower Circf	0.554 **	0.112 *	0.460 **	0.580 **	0.700 **	0.723 **		
	0.351 **	0.764 **	0.233 **	0.394 **	0.611 **	0.596 **		
Upper Circf	0.497 **	0.105 *	0.422 **	0.558 **	0.639 **	0.688 **	0.882 **	
	0.296 **	0.795 **	0.193 **	0.394 **	0.556 **	0.581 **	0.832 **	
Tine Num	0.357 **	0.103 *	0.223 **	0.471 **	0.670 **	0.594 **	0.627 **	0.676 **
	0.244 **	0.611 **	0.169*	0.392 **	0.629 **	0.570 **	0.598 **	0.671 **

Probability levels as follows: * for *p* < 0.05; ** for *p* < 0.01.

**Table 4:** General Linear Model of antler weight, beam length, upper beam circumference, and number of tines, and the significant factors influencing them. Eta^2^ shows the weight of each factor in the model.

Antler Weight			
Variable	Coefficient	SE	Eta^2^
R^2^ corrected			0.817
Intercept	−11.67	±0.9 ***	0.473
Beam length	0.6	±0.01 ***	0.193
Burr (section)	0.15	±0.05 **	0.054
Midbeam Tine Length	0.09	±0.01 ***	0.206
Upper Beam Circf.	0.39	±0.04 ***	0.297
**Beam Length**			
**Variable**	**Coefficient**	**SE**	**Eta^2^**
R^2^ corrected			0.394
Intercept	78.45	±4.60 ***	0.607
Antler weight	2.84	±0.41 ***	0.206
Age	1.02	±0.41 *	0.033
Brow (1st) Tine Length	0.20	±0.1 *	0.022
Upper Beam Circf	−1.41	±0.34 ***	0.085
**Upper Beam Circfumference**			
**Variable**	**Coefficient**	**SE**	**Eta^2^**
R^2^ corrected			0.338
Intercept	3.13	±1.21 **	0.031
Burr	0.48	±0.05 ***	0.338
**Number of Tines**			
**Variable**	**Coefficient**	**SE**	**Eta^2^**
R^2^ corrected			0.556
Intercept	−2.09	±2.80	0.003
Beam length	−0.08	±0.03 *	0.030
Midbeam Tine length	0.32	±0.05 ***	0.200
Upper Beam Circfumference	1.02	±0.13 ***	0.230

Probability levels as follows: * for *p* < 0.05; ** for *p* < 0.01; *** for *p* < 0.001.

## Data Availability

The data presented in this study are available on request from the corresponding author.

## Abbreviations

The following abbreviations are used in this manuscript:

LIAC	Latvia International Antler Competition
UCLM	Universidad de Castilla-La Mancha
GLM	Generalized Linear Model
